# Personality States and Cognitive Ability and Variability in Older Adults

**DOI:** 10.1093/geroni/igaf122.278

**Published:** 2025-12-31

**Authors:** Winkie Ma, Adam Nissen, Eileen Graham, Zoë Hawks, Emorie Beck

**Affiliations:** University of California, Davis, Davis, California, United States; University of California, Davis, Davis, California, United States; Northwestern University, Evanston, Illinois, United States; McLean Hospital, Boston, Massachusetts, United States; University of California, Davis, Davis, California, United States

## Abstract

Psychological factors, like personality, are particularly promising behavioral indicators of AD/ADRD to target as risk-modifying treatments. Personality traits are typically defined as characteristic patterns of thinking, feeling, and behaving, which are expressed in everyday life as personality states. Prior work linking personality to cognition has largely focused on how broad, between-person differences in personality traits are associated with broad differences in cognitive function and decline, ignoring personality states and the within-person processes that unfold on shorter time scales. The present study aims to extend prior work by investigating associations between Big Five states and momentary cognitive function (i.e. momentary levels of cognitive function) and variability using an ecological momentary assessment (EMA) study of middle and older adults between 45 and 74. At baseline, 200 (Mean age = 55; 50.1% female, total obs. = 12,431) participants responded to five surveys per day for 20 days capturing their cognitive function, contextual factors, and Big Five personality states. Using mixed-effects location scale models, we simultaneously predicted momentary cognitive levels (location) and cognitive variability (scale) from fluctuations in personality states. In line with prior work on personality traits, we expected that Conscientiousness and Neuroticism states would impact both cognitive levels and variability, on average, across the sample. However, we also expected individual differences in the degree to which each was associated with cognitive function and variability across people, which would signal differences in vulnerability to personality states that could be targets for intervention.

